# Parental metabolic syndrome and elevated liver transaminases are risk factors for offspring, even in children and adolescents with a normal body mass index

**DOI:** 10.3389/fnut.2023.1166244

**Published:** 2023-10-24

**Authors:** Kyungchul Song, Juyeon Yang, Hye Sun Lee, Jun Suk Oh, Sujin Kim, Myeongseob Lee, Junghwan Suh, Ahreum Kwon, Ho-Seong Kim, Hyun Wook Chae

**Affiliations:** ^1^Department of Pediatrics, Yonsei University College of Medicine, Seoul, Republic of Korea; ^2^Biostatistics Collaboration Unit, Yonsei University College of Medicine, Seoul, Republic of Korea; ^3^Department of Pediatrics, Konyang University College of Medicine, Daejeon, Republic of Korea

**Keywords:** metabolic syndrome, non-alcoholic fatty liver disease, child, adolescent, parent–child relations

## Abstract

**Introduction:**

The parent–child correlation in metabolic syndrome (MetS) and elevated transaminases is sparsely researched. We assessed the correlation of parental MetS and elevated transaminase status with these conditions in their children.

**Methods:**

Data of 4,167 youths aged 10–18 years were analyzed in a population-based survey, and the parental characteristics were stratified by the presence or absence of MetS or alanine aminotransferase (ALT) elevation in their children. The prevalence of these conditions in children was analyzed according to their parents’ status. Logistic regression analyses were performed with MetS and ALT elevation in youth as the dependent variables.

**Results:**

The proportions of MetS and ALT elevation were higher in parents of children with MetS and ALT elevation than in those without, even among youths without obesity. In logistic regression analyses, age, body mass index–standard deviation score (BMI–SDS), and ALT elevation were positively associated with MetS, whereas age, male sex, BMI–SDS, protein intake, and MetS were positively associated with ALT elevation. Higher protein intake was related to ALT elevation, whereas metabolic components and nutritional factors were closely related in parents and their children. Odds ratios (OR) of ALT elevation for MetS was 8.96 even after adjusting nutritional factors in the children. The OR was higher for ALT elevation in the children of parents with MetS and ALT elevation compared to those without. ORs for MetS and ALT elevation in the children of parents with MetS were higher than those of children of parents without MetS, even after adjusting for nutritional intake. ORs for ALT elevation were higher in the children of parents with ALT elevation than those without, even after adjusting for nutritional intake and BMI of parents as well as the nutritional intake, age, sex, and BMI–SDS of the children.

**Conclusion:**

MetS and elevated liver transaminase statuses in children were associated with those of their parents even after adjusting for nutritional factors, and the relationships were more prominent in the youth without obesity.

## 1. Introduction

Metabolic syndrome (MetS) constitutes a cluster of risk factors for cardiovascular disease and increases the risk of atherosclerotic cardiac disease; moreover, childhood MetS increases the risk of adult MetS (1, 2). A systematic review showed that, among children and adolescents, MetS prevalence ranged from 0.3% (in Columbia) to 26.4% (in Iran) (3). The overall median prevalence was 3.8%, and it was 5 and 5.4% in Saudi Arabia and the United States, respectively. A study in the United States reported a decline in the prevalence of MetS among youth from 7.3% in 1988–1994 to 6.5% in 2003–2006 (4). In contrast, a Korean study showed that the prevalence of MetS increased from 1.5% in 2008 to 3.2% in 2017 among youth (1). In adults, obesity, overnutrition, and low lean mass are risk factors for MetS (5). In a cohort study, MetS in children was correlated with MetS in parents (6).

Nonalcoholic fatty liver disease (NAFLD) is a chronic liver disease characterized by excessive hepatic fat accumulation in a spectrum that ranges from nonalcoholic steatohepatitis, hepatic fibrosis, to idiopathic cirrhosis, without significant alcohol consumption (7–9). Among patients with nonalcoholic steatohepatitis, the annual cumulative incidence of liver cirrhosis is 0.25–3.2%; of this, 0.3–2.6% of cases with cirrhotic nonalcoholic steatohepatitis progress to hepatocellular carcinoma per year (10). In a global study, the prevalence of NAFLD was 30%, whereas in a pediatric study, the prevalence increased from 3.9% in 1988–1994 to 10.7% in 2007–2010 (11, 12).

In the pathogenesis of MetS and NAFLD, insulin resistance plays a key role (13–15). Furthermore, obesity, unhealthy dietary habits, and MetS are risk factors for NAFLD progression in adults (10, 16). A meta-analysis showed an association of NAFLD with a twofold increased risk of MetS in adults (17). Based on the above-described relationships, a novel concept—metabolic-associated fatty liver disease (MAFLD)—has been posited (18). MAFLD is identified by its association with metabolic dysregulation, irrespective of alcohol consumption. Therefore, addressing pediatric MAFLD and NAFLD is important to prevent severe hepatic as well as metabolic disorders in adulthood (18, 19). However, research on the risk factors for pediatric MetS and MAFLD or NAFLD in association with parental MetS and MAFLD or NAFLD remains limited.

In this population-based study, we aimed to investigate the association of pediatric MetS and elevated liver transaminase levels with these conditions in the parents. The study’s objectives were to investigate: (1) the relationship between MetS and elevated liver transaminases in youths; (2) the risk factors of MetS and elevated liver transaminases in youths; and (3) the association of MetS and elevated liver transaminase levels in parents with these conditions in children.

## 2. Materials and methods

### 2.1. Study design and participants

This cross-sectional study analyzed data from the Korea National Health and Nutrition Examination Survey (KNHANES), a national population-based survey conducted by the Korea Centers for Disease Control and Prevention based on the National Health Promotion Act, that were obtained between 2007 and 2019. The KNHANES was conducted in 1998, 2001, 2005, and annually since 2007 using a rolling sampling survey method. The cross-sectional survey includes approximately 10,000 participants each year as a cohort and investigates data on health behaviors and clinical examination, including anthropometric and laboratory analysis-based information, socioeconomic status, nutritional status, the presence of chronic disease, and quality of life. The survey involved a two-step stratified sampling method that used sampling units and households as the primary and secondary sampling units, respectively. In total, 200 and 192 primary sampling units (PSU) per year were drawn from approximately 200,000 geographically defined PSU nationwide in KNHANES 2007–2009 and KNHANES 2010–2019, respectively. On average, a PSU comprises 60 households, and 20 final target households were sampled for each PSU using systematic sampling; individuals who were ≥ 1-year-old were targeted from the selected households.

**Figure 1:**
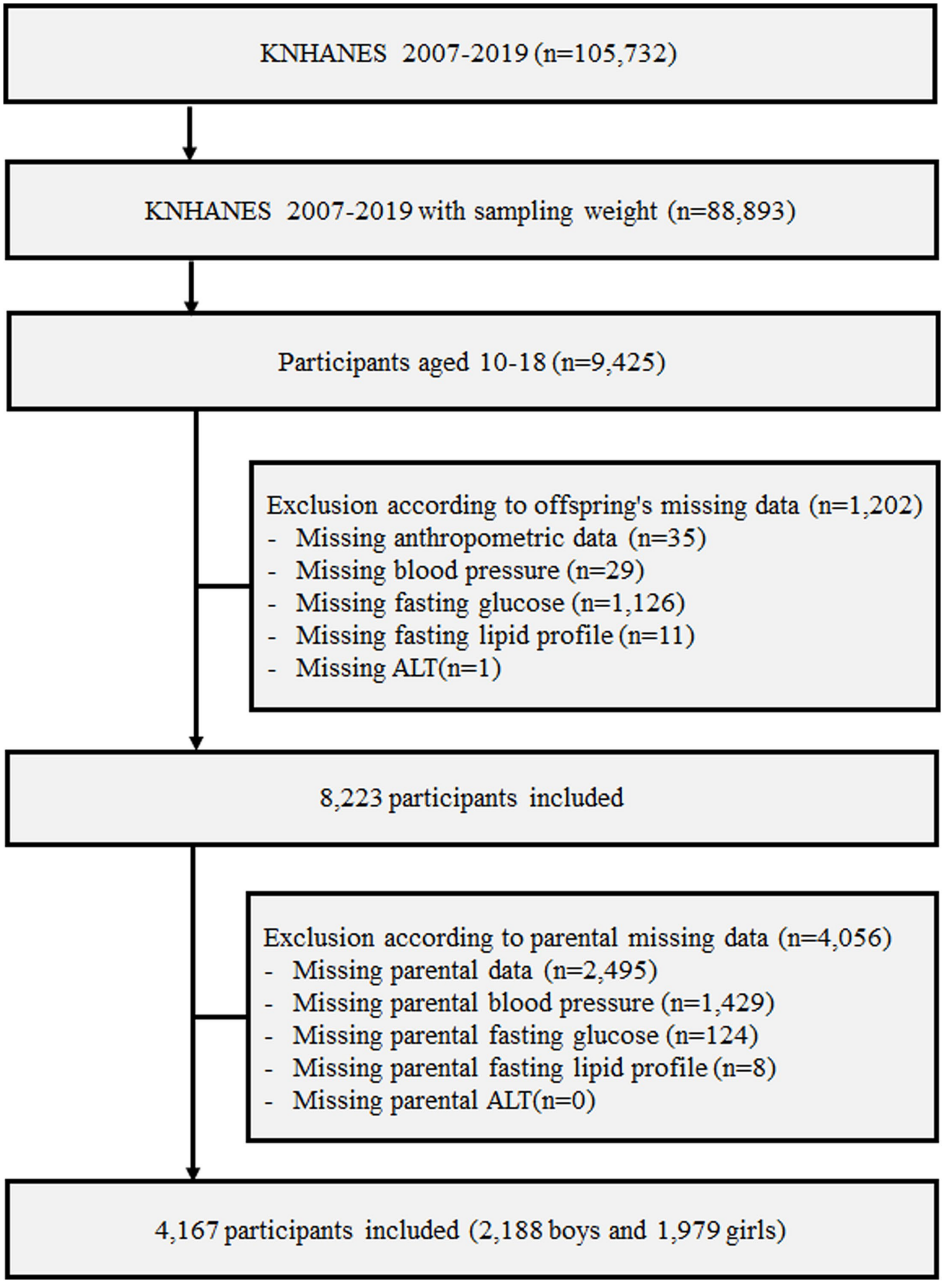
Design and flowchart of study population. KNHANES, Korea National Health and Nutrition Examination Survey; ALT, alanine aminotransferase.

From among children and adolescents as well as their parents who participated in KNHANES 2007–2019, individuals whose anthropometric, blood pressure, fasting glucose and lipid, and alanine aminotransferase (ALT) data were missing were excluded. Finally, data from 4,167 children and adolescents as well as their respective parents were analyzed in this study; details of the participant screening and selection are presented in a flowchart in Figure 1.

To ensure accurate representation of the Korean population and minimize selection bias, a multistage-stratified systematic sampling design was used wherein the sample weights were constructed for sample participants by accounting for the complex survey design, survey non-response, and post-stratification. The weights based on the inverse of selection probabilities and response rates were modified by adjusting them to the sex- and age-specific Korean populations (20).

### 2.2. Study variables

Data on sex, age, and anthropometric measurements were recorded. Height was measured using a portable stadiometer (range, 850–2060 mm; Seriter, Holtain Ltd., Crymych, UK), and weight was measured using a calibrated balance beam scale (Giant 150N; HANA, Seoul, Republic of Korea). Body mass index (BMI) was calculated as the weight (kg) divided by the height (in meters) squared (m^2^). Waist circumference (WC) was measured at the narrowest point between the lower borders of the rib cage and the iliac crest at the end of normal expiration. Height, weight, and BMI were presented as standard deviation scores (SDS) based on the 2017 Korean National Growth Charts (21). Based on their BMI, children were categorized as normal (<85th percentile), overweight (85th to <95th percentile), or obese (≥95th percentile). Central obesity was defined as a WC >90th percentile using Korean waist reference data (22).

Information on food intake was obtained via face-to-face interview (20). The food frequency questionnaire consisted of 63 food items that are important sources of energy and nutrients. The food intake questionnaire was created as an open-ended survey to report different meals and foods, and utilized the 24 h recall method with different measurement tools.

### 2.3. Laboratory analysis

Blood samples were collected from the antecubital vein after at least 8 h of fasting and were subsequently processed and immediately refrigerated. Serum levels of fasting glucose, total cholesterol, high-density lipoprotein cholesterol (HDL-C), triglycerides, aspartate aminotransferase (AST), and ALT were measured using Labospect 008AS (Hitachi, Tokyo, Japan). Low-density lipoprotein cholesterol (LDL-C) was calculated using the Friedewald formula (LDL-C = total cholesterol − [HDL-C + (triglycerides/5)]). The triglycerides/5 were used for serum samples with triglyceride values ≤400 mg/dL, whereas samples with triglyceride levels >400 mg/dL were considered as missing values in the analysis (23). “ALT elevation” was defined as an increased ALT level (>26, > 22, >30, and > 19 IU/L for boys, girls, men, and women, respectively) without hepatitis B virus infection or significant alcohol consumption, which was defined as the intake of >140 and > 70 g/week alcohol in men and women, respectively (8, 16, 24, 25).

### 2.4. Statistical analysis

All statistical analyses were performed using SAS (version 9.4; SAS Institute, Cary, NC, USA) for the complex sampling design, with clustering, stratification, and unequal weighting of the KNHANES sample and considering sampling weights to present data on the representative Korean youth population and to overcome limitations conferred by the sampling survey design. All categorical variables are presented as weighted percentages with standard errors, whereas continuous variables are presented as weighted means with standard errors. Participants were stratified into subgroups based on the presence of MetS and ALT elevation, and the proportion of participants with MetS or ALT elevation and the characteristics of their parents were analyzed. Moreover, the prevalence of MetS and ALT elevation was analyzed in subgroups stratified by BMI and the presence of parental MetS and ALT elevation. Intergroup differences were ascertained using the Student’s *t*-test for continuous variables and the Rao–Scott chi-square test for categorical variables. To clarify the risk factors for pediatric MetS and ALT elevation, logistic regression analyses were performed with MetS and ALT elevation in youth as the dependent variables. Furthermore, to clarify the association of MetS and ALT elevation between children and their parents, logistic regression analyses were performed with the MetS and ALT elevation of the children and parents as dependent and independent variables, respectively. Linear regression analyses were performed with each component of MetS, liver enzymes, and nutritional intake of parents and children as independent and dependent variables, respectively. A *p*-value < 0.05 was considered statistically significant.

## 3. Results

### 3.1. Association of MetS with elevated liver transaminases among youths

Table 1 shows the baseline characteristics of participants according to the presence of MetS and ALT elevation. Compared to the youth without MetS, the youth with MetS had higher values for age, BMI SDS, AST, ALT, and the proportion of obesity and ALT elevation. Each component of MetS, including WC, blood pressure, triglycerides, and glucose, as well as the proportion of central obesity and MetS, were higher in the youth with ALT elevation than in those without ALT elevation. Furthermore, compared to the youth without ALT elevation, age, BMI SDS, intake of energy and protein, and the proportion of male sex and obesity were higher in the youths with ALT elevation.

**Table 1:** Baseline characteristics of offspring according to MetS and ALT elevation.

	Total (*n* = 4,167)	Non-MetS (*n* = 4,093)	MetS (*n* = 74)	*p*	Normal ALT (*n* = 3,790)	ALT elevation (*n* = 377)	*p*
Sex (male), %	53.07 (0.87)	52.86 (0.87)	62.97 (6.12)	0.112	50.86 (0.90)	73.86 (2.48)	<0.001
Age, y	14.23 (0.05)	14.22 (0.05)	14.96 (0.30)	0.016	14.18 (0.050)	14.688 (0.16)	0.002
Height SDS	0.22 (0.02)	0.22 (0.02)	0.36 (0.16)	0.377	0.22 (0.022)	0.26 (0.06)	0.543
Weight SDS	0.08 (0.02)	0.04 (0.02)	2.18 (0.09)	<0.001	−0.03 (0.023)	1.18 (0.08)	<0.001
BMI, kg/m^2^	20.78 (0.08)	20.62 (0.07)	28.50 (0.40)	<0.001	20.33 (0.067)	24.94 (0.27)	<0.001
BMI SDS	−0.04 (0.03)	−0.09 (0.03)	2.43 (0.10)	<0.001	−0.18 (0.024)	1.28 (0.08)	<0.001
BMI percentile, %				<0.001			<0.001
Normal	79.61 (0.766)	81.18 (0.754)	3.93 (2.349)		83.54 (0.714)	42.64 (2.915)	
Overweight	9.93 (0.525)	9.90 (0.529)	11.33 (4.400)		9.12 (0.516)	17.50 (2.293)	
Obesity	10.46 (0.618)	8.92 (0.583)	84.74 (4.840)		7.34 (0.537)	39.86 (2.937)	
WC, cm	70.01 (0.2)	69.57 (0.19)	90.89 (0.89)	<0.001	68.774 (0.178)	81.609 (0.732)	<0.001
Central obesity, %	10.22 (0.578)	8.35 (0.527)	100.00 (0.000)	<0.001	7.29 (0.514)	37.76 (2.853)	<0.001
Systolic BP, mmHg	107.28 (0.21)	107.04 (0.21)	118.45 (1.52)	<0.001	106.66 (0.211)	113.06 (0.659)	<0.001
Diastolic BP, mmHg	66.4 (0.17)	66.27 (0.17)	72.98 (1.37)	<0.001	66.07 (0.178)	69.52 (0.551)	<0.001
Total cholesterol, mg/dL	160.16 (0.53)	159.82 (0.54)	176.42 (3.92)	<0.001	158.81 (0.533)	172.90 (1.948)	<0.001
HDL-C, mg/dL	51.19 (0.21)	51.47 (0.21)	37.89 (0.61)	<0.001	51.53 (0.217)	48.00 (0.635)	<0.001
Triglycerides, mg/dL	85.51 (1)	83.33 (0.93)	190.92 (10.24)	<0.001	82.05 (0.919)	118.13 (4.630)	<0.001
LDL-C, mg/dL	92.59 (0.46)	92.30 (0.46)	106.84 (3.25)	<0.001	91.40 (0.453)	103.82 (1.792)	<0.001
Glucose, mg/dL	90.15 (0.18)	90.08 (0.18)	93.36 (1.14)	0.004	89.97 (0.187)	91.79 (0.627)	0.005
AST, IU/L	19.47 (0.17)	19.29 (0.16)	27.86 (2.98)	0.004	18.13 (0.088)	32.02 (1.305)	<0.001
ALT, IU/L	15.79 (0.33)	15.31 (0.31)	39.00 (5.37)	<0.001	12.34 (0.085)	48.32 (2.619)	<0.001
ALT elevation, %	9.59 (0.555)	8.83 (0.536)	46.33 (6.704)	<0.001			
MetS, %	2.03 (0.266)				1.21 (0.220)	9.81 (1.803)	<0.001
Energy intake, kcal	2151.62 (16.91)	2152.97 (17.08)	2086.81 (125.44)	0.602	2137.61 (17.28)	2283.57 (69.02)	0.041
Carbohydrate intake, g	325.79 (2.51)	325.89 (2.55)	321.16 (18.18)	0.798	324.341 (2.604)	339.47 (9.970)	0.146
Protein intake, g	78.39 (0.80)	78.42 (0.81)	76.74 (5.40)	0.758	77.42 (0.786)	87.46 (3.360)	0.003
Fat intake, g	57.77 (0.71)	57.90 (0.72)	51.66 (4.54)	0.172	57.48 (0.730)	60.51 (2.647)	0.269

Continuous variables are presented as the mean (standard error) and categorical data as the percentage (standard error).

MetS, metabolic syndrome; ALT, alanine aminotransferase; SDS, standard deviation score; BMI, body mass index; WC, waist circumference; BP, Blood pressure; HDL-C, high-density lipoprotein cholesterol; LDL-C, low-density lipoprotein cholesterol; AST, aspartate aminotransferase; ALT, alanine aminotransferase.

In univariable logistic regression analyses, the odds ratio (OR; 95% confidence interval [CI]) for MetS in age, BMI SDS, and ALT elevation were 1.13 (1.02–1.25), 1.52 (0.90–2.55), 4.01 (3.31–4.85), and 8.91 (5.16–15.38), respectively (Supplementary Table 1). The OR (95% CI) for ALT elevation in age, male sex, BMI SDS, protein intake, and MetS were 1.08 (1.03–1.14), 2.73 (2.10–3.54), 2.35 (2.11–2.61), 1.05 (1.02–1.08), and 8.91 (5.16–15.38). After adjusting nutrition factors, including carbohydrate, protein, and fat, the OR (95% CI) of MetS for ALT elevation was 8.98 (5.22–15.46) and that of ALT elevation for MetS was 8.96 (5.20–15.44). However, these relationships were not significant after adjusting for age, sex, BMI–SDS, and nutritional factors.

### 3.2. Parental characteristics in subgroups stratified by the children’s MetS and elevated liver transaminase status

Table 2 shows the parental characteristics according to the MetS and ALT elevation status of the children. Compared to the fathers and mothers of the youth without MetS, the parents of the youth with MetS had a higher BMI, WC, and proportion of MetS. Similarly, parents of youth with ALT elevation had higher values of age, BMI, WC, AST, ALT, and the proportion of obesity and ALT elevation compared to the parents of youth without ALT elevation. Moreover, fathers of youth with ALT elevation had higher systolic and diastolic blood pressures and glucose levels compared to fathers of youth without ALT elevation.

**Table 2:** Characteristics of parents according to offspring’s MetS and ALT elevation.

	Non-MetS (*n* = 4,093)	MetS (*n* = 74)	*p*	Normal ALT (*n* = 3,790)	ALT elevation (*n* = 377)	*p*
Father						
Age, y	46.17 (0.10)	47.07 (0.55)	0.111	46.11 (0.10)	46.93 (0.31)	0.009
BMI, kg/m^2^	24.72 (0.07)	25.80 (0.42)	0.010	24.66 (0.07)	25.46 (0.18)	<0.001
BMI percentile, %			0.121			<0.001
Normal	28.84 (1.009)	17.10 (4.693)		29.47 (1.05)	20.39 (2.39)	
Overweight	26.84 (0.996)	27.32 (6.443)		27.42 (1.04)	21.42 (2.43)	
Obesity	44.33 (1.100)	55.59 (6.760)		43.11 (1.13)	58.19 (3.03)	
WC, cm	85.94 (0.19)	89.20 (1.05)	0.002	85.81 (0.19)	87.96 (0.49)	<0.001
Central obesity, %	31.18 (1.073)	38.34 (6.446)	0.247	30.52 (1.08)	38.90 (3.10)	0.005
Systolic BP, mmHg	118.87 (0.31)	120.13 (1.83)	0.491	118.66 (0.33)	121.05 (0.84)	0.006
Diastolic BP, mmHg	81.29 (0.23)	83.60 (1.41)	0.102	81.16 (0.24)	83.061 (0.63)	0.003
HDL-C, mg/dL	46.53 (0.24)	43.23 (1.21)	0.007	46.54 (0.25)	45.72 (0.60)	0.185
Triglycerides, mg/dL	176.18 (3.41)	250.16 (43.80)	0.092	176.40 (3.53)	189.83 (8.77)	0.122
Glucose, mg/dL	101.04 (0.49)	106.73 (5.35)	0.288	100.65 (0.48)	105.86 (1.85)	0.004
AST, IU/L	25.79 (0.58)	25.75 (1.37)	0.983	25.45 (0.48)	28.9 (1.82)	0.023
ALT, IU/L	29.60 (1.01)	32.54 (2.75)	0.313	25.45 (0.48)	28.90 (1.82)	0.023
ALT elevation, %	19.14 (0.867)	23.29 (5.646)	0.437	18.60 (0.87)	25.12 (2.52)	0.005
MetS, %	21.96 (0.953)	34.74 (6.298)	0.021	28.95 (0.78)	36.37 (3.42)	0.008
Energy intake, kcal	2525.57 (21.37)	2519.05 (120.89)	0.957	2522.31 (22.06)	2554.88 (59.88)	0.598
Carbohydrate intake, g	358.37 (2.88)	356.97 (17.03)	0.935	358.51 (2.99)	356.72 (7.47)	0.817
Protein intake, g	91.23 (1.00)	91.75 (6.47)	0.935	91.16 (1.02)	92.02 (2.93)	0.770
Fat intake, g	55.45 (0.83)	52.22 (5.44)	0.552	55.05 (0.84)	58.54 (2.61)	0.183
Mother						
Age, y	43.20 (0.09)	43.77 (0.50)	0.261	43.15 (0.10)	43.84 (0.28)	0.016
BMI, kg/m^2^	23.29 (0.07)	25.19 (0.50)	<0.001	23.24 (0.07)	24.19 (0.21)	<0.001
BMI percentile, %			<0.001			<0.001
Normal	51.47 (1.180)	29.14 (6.019)		51.97 (1.21)	42.06 (3.02)	
Overweight	24.23 (1.030)	21.09 (5.384)		24.44 (1.05)	21.65 (2.61)	
Obesity	24.29 (0.955)	49.77 (6.772)		23.60 (0.98)	36.30 (2.85)	
WC, cm	77.34 (0.19)	82.23 (1.31)	<0.001	77.23 (0.19)	79.45 (0.58)	<0.001
Central obesity, %	33.29 (1.104)	58.06 (6.790)	<0.001	32.62 (1.12)	44.92 (3.03)	<0.001
Systolic BP, mmHg	111.11 (0.32)	117.79 (2.08)	0.001	111.08 (0.33)	112.84 (0.93)	0.065
Diastolic BP, mmHg	73.90 (0.22)	77.10 (1.27)	0.012	73.89 (0.23)	74.67 (0.61)	0.216
HDL-C, mg/dL	54.26 (0.28)	49.92 (2.03)	0.032	54.12 (0.29)	54.71 (0.72)	0.422
Triglycerides, mg/dL	102.67 (1.43)	127.87 (13.46)	0.061	102.89 (1.50)	105.96 (3.83)	0.436
Glucose, mg/dL	94.78 (0.46)	98.79 (2.46)	0.107	94.69 (0.48)	96.53 (1.22)	0.145
AST, IU/L	19.16 (0.21)	19.71 (1.18)	0.645	19 (0.22)	20.81 (0.51)	<0.001
ALT, IU/L	16.60 (0.27)	18.30 (2.10)	0.419	28.95 (0.78)	36.3 7 (3.42)	0.008
ALT elevation, %	17.59 (0.880)	18.87 (4.951)	0.792	16.64 (0.871)	26.86 (2.881)	<0.001
MetS, %	9.70 (0.629)	26.38 (6.096)	<0.001	9.72 (0.652)	13.04 (1.971)	0.067
Energy intake, kcal	1761.46 (15.00)	1699.75 (113.23)	0.584	1760.36 (15.52)	1758.78 (42.50)	0.971
Carbohydrate intake, g	282.16 (2.50)	268.89 (17.07)	0.434	282.01 (2.58)	280.77 (7.26)	0.867
Protein intake, g	63.59 (0.67)	63.98 (5.71)	0.945	63.49 (0.69)	64.66 (2.07)	0.576
Fat intake, g	39.21 (0.61)	37.15 (3.74)	0.583	39.16 (0.63)	39.231 (1.54)	0.967

Continuous variables are presented as the mean (standard error) and categorical data as the percentage (standard error).

MetS, metabolic syndrome; ALT, alanine aminotransferase; BMI, body mass index; WC, waist circumference; BP, Blood pressure; HDL-C, high-density lipoprotein cholesterol; LDL-C, low-density lipoprotein cholesterol; AST, aspartate aminotransferase; ALT, alanine aminotransferase.

### 3.3. Characteristics of youth according to parental MetS and elevated liver transaminase status

Table 3 shows the characteristics of the youth according to the parents’ MetS and ALT elevation status. The progeny of parents with MetS had higher BMI SDS, WC, systolic and diastolic blood pressures, glucose levels, and proportions of obesity, central obesity, and MetS compared to the progeny of parents without MetS. Moreover, AST, ALT, and fat intake levels were higher in the progeny of parents with MetS than in those of parents without MetS. The children of parents with ALT elevation had higher WC, ALT level, and proportions of obesity and ALT elevation compared to those of parents without ALT elevation.

**Table 3:** Characteristics of the offspring according to parents’ MetS and ALT elevation.

	Parents without MetS (*n* = 2,939)	Parents with MetS (*n* = 1,212)	*p*	Parents without ALT elevation (*n* = 2,821)	Parents with ALT elevation (*n* = 1,346)	*p*
BMI SDS	−0.18 (0.03)	0.31 (0.05)	<0.001	−0.065 (0.031)	0.023 (0.049)	0.132
BMI percentile, %			<0.001			0.045
Normal	83.41 (0.837)	70.32 (1.642)		80.87 (0.89)	77.05 (1.51)	
Overweight	8.48 (0.603)	13.48 (1.115)		9.63 (0.62)	10.52 (0.98)	
Obesity	8.11 (0.638)	16.21 (1.366)		9.50 (0.71)	12.43 (1.23)	
WC	68.82 (0.22)	72.86 (0.41)	<0.001	69.644 (0.238)	70.741 (0.366)	0.012
Central obesity, %	7.82 (0.62)	16.20% (1.31)	<0.001	9.82 (0.708)	11.02 (1.069)	0.352
Systolic BP	106.78 (0.25)	108.39 (0.39)	0.015	107.122 (0.252)	107.585 (0.363)	0.287
Diastolic BP	66.10 (0.20)	67.03 (0.33)	0.010	66.357 (0.212)	66.494 (0.302)	0.708
Glucose, mg/dL	89.82 (0.19)	90.99 (0.42)	<0.001	89.968 (0.186)	90.511 (0.371)	0.172
HDL-C, mg/dL	51.70 (0.24)	49.97 (0.40)	0.022	51.474 (0.253)	50.616 (0.349)	0.042
Triglycerides, mg/dL	84.06 (1.18)	88.89 (1.77)	0.398	85.005 (1.265)	86.552 (1.627)	0.455
AST, IU/L	19.34 (0.15)	19.73 (0.44)	0.002	19.326 (0.215)	19.749 (0.260)	0.212
ALT, IU/L	14.88 (0.28)	17.78 (0.88)	<0.001	15.057 (0.399)	17.287 (0.597)	0.002
ALT elevation, %	8.33 (0.623)	12.46 (1.115)	0.658	8.22 (0.643)	12.40 (1.032)	<0.001
MetS %	1.44 (0.275)	3.53 (0.642)	<0.001	1.98 (0.330)	2.13 (0.464)	0.800
Energy intake, kcal	2144.51 (19.17)	2162.27 (34.77)	0.654	2137.937 (20.301)	2179.452 (31.480)	0.273
Carbohydrate intake, g	324.75 (2.85)	327.41 (5.17)	0.580	324.275 (3.066)	328.882 (4.327)	0.384
Protein intake, g	78.01 (0.91)	79.03 (1.61)	0.850	77.642 (0.927)	79.905 (1.606)	0.228
Fat intake, g	57.64 (0.84)	57.93 (1.32)	<0.001	57.217 (0.853)	58.897 (1.338)	0.296

Continuous variables are presented as the mean (standard error) and categorical data as the percentage (standard error).

MetS, metabolic syndrome; ALT, alanine aminotransferase; BMI, body mass index; SDS, standard deviation score; WC, waist circumference; BP, Blood pressure; HDL-C, high-density lipoprotein cholesterol; AST, aspartate aminotransferase; ALT, alanine aminotransferase.

**Figure 2:**
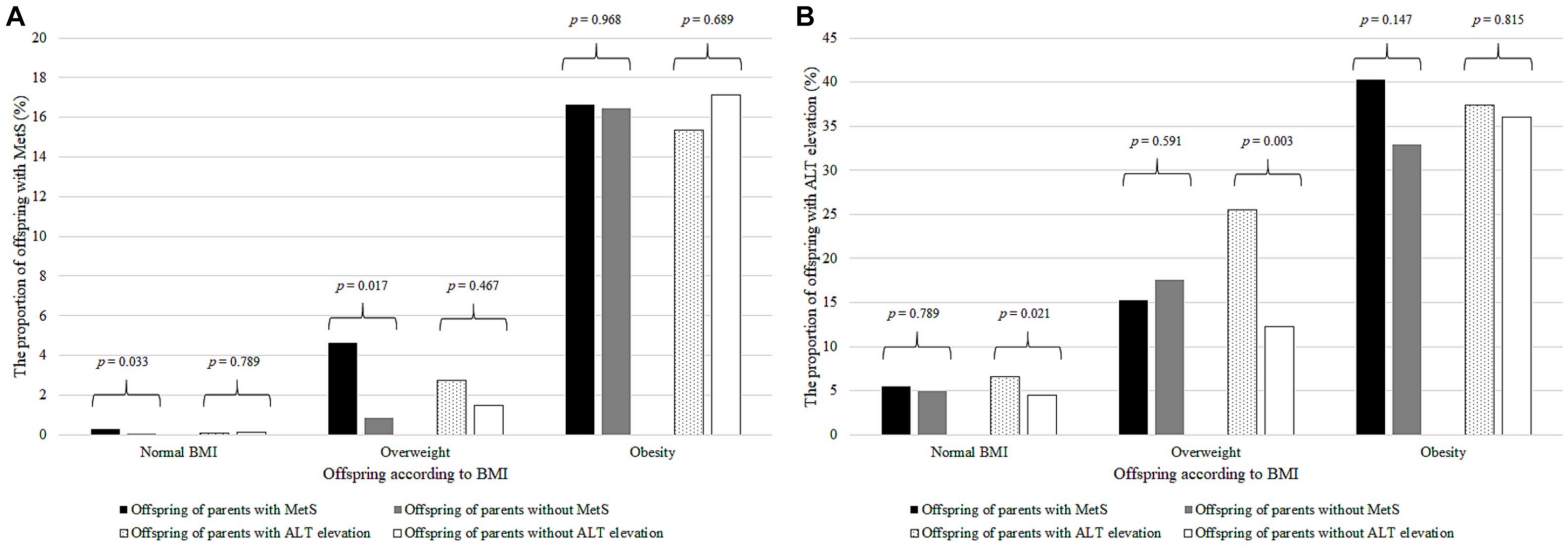
The proportion of offspring with MetS or ALT elevation according to BMI and parental MetS and ALT elevation. **(A)** The proportion of offspring with MetS according to BMI and parental MetS and ALT elevation. The black bar represents the offspring with MetS of the parents with MetS; gray bar, parents without MetS; dotted bar, parents with ALT elevation; and the white bar, parents without ALT elevation. The number on the bar is the *p*–value of Student’s *t*–test. **(B)** Proportion of offspring with ALT elevation according to BMI and parental MetS and ALT elevation. The black bar represents the offspring with ALT elevation of the parents with MetS; gray bar, those of the parents without MetS; dotted bar, those of the parents with ALT elevation; and the white bar, those of the parents without ALT elevation. The number on the bar is the *p*–value of Student’s *t*–test. MetS, metabolic syndrome; ALT, alanine aminotransferase; BMI, body mass index.

Among youth with normal BMI or overweight, the proportion of children with MetS was higher in the progeny of parents with MetS than in those without (*p* = 0.033 for normal BMI and *p* = 0.017 for overweight; Figure 2A), whereas the proportion of offspring with ALT elevation was higher in the offspring of parents with ALT elevation than in those without (*p* = 0.021 for normal BMI and *p* = 0.003 for overweight; Figure 2B). However, these relationships were not significant among the youth with obesity.

Supplementary Table 2 shows the characteristics of the children of parents without MetS or ALT elevation. The children of parents with MetS had higher BMI SDS, WC, systolic and diastolic blood pressures, glucose, triglycerides, ALT, and proportions of obesity, central obesity, and ALT elevation compared to those without MetS. Compared to the children of parents without ALT elevation, those whose parents had ALT elevation had higher BMI SDS, WC, systolic and diastolic blood pressures, AST, ALT, protein intake, and proportions of obesity, central obesity, and MetS.

### 3.4. Linear regression of children’s and parents’ metabolic components and nutrition

Linear regression analyses showed that BMI, each component of MetS, AST, ALT, and nutritional factors of the father and mother were postively associted with those of the children, except for the glucose level between the mother and child (Supplementary Table 3). Among the factors, the association with WC was the strongest.

### 3.5. OR of parental and children’s MetS and elevated liver transaminase levels

The children of fathers and mothers with MetS had a higher OR (2.26, 95% CI 1.27–4.04 and 3.88, 95% CI 1.99–7.56, respectively) for MetS compared to the children of parents without MetS; the OR was highest in the children whose both parents had MetS (OR 4.97, 95% CI 1.97–12.53) (Table 4). Additionally, a relationship between the parental MetS and the child’s MetS and ALT elevation status was identified even after adjusting for the parent’s and progeny’s nutritional intake, including carbohydrate, protein, and fat. Moreover, compared to the children whose parents did not have ALT elevation, the OR for ALT elevation was higher in the youths whose father, mother, or both parents had ALT elevation, and this effect persisted even after adjusting for the children’s age, sex, BMI–SDS, nutritional intake, and parental BMI and nutritional intake status.

**Table 4:** ORs and 95% CIs for MetS and ALT elevation in offspring by parents’ MetS and ALT elevation.

		OR (95% CI) for offspring’s MetS					
	No. (%) of offspring with MetS	Unadjusted	*p*	Adjusted^a^	*p*	Adjusted^b^	*p*
Parents without MetS	36/2939 (1.44)	1.00		1.00		1.00	
Father with MetS	27/920 (3.19)	2.26 (1.27–4.04)	0.006	2.26 (1.26–4.06)	0.006	1.50 (0.57–3.91)	0.408
Mother with MetS	19/431 (5.35)	3.88 (1.99–7.56)	<0.001	3.85 (1.99–7.46)	<0.001	2.43 (0.73–8.05)	0.147
Both parents with MetS	8/139 (6.74)	4.97 (1.97–12.53)	<0.001	4.96 (2.00–12.32)	<0.001	4.12 (0.59–28.94)	0.155
Parents without ALT elevation	48/2821 (1.98)	1.00		1.00		1.00	
Father with ALT elevation	17/790 (2.46)	1.25 (0.65–2.38)	0.503	1.24 (0.64–2.41)	0.525	1.03 (0.47–2.26)	0.946
Mother with ALT elevation	15/724 (2.18)	1.10 (0.57–2.12)	0.778	1.12 (0.58–2.15)	0.744	0.79 (0.37–1.67)	0.530
Both parents with ALT elevation	6/168 (3.98)	2.05 (0.80–5.25)	0.135	2.13 (0.83–5.43)	0.114	1.22 (0.43–3.42)	0.709

		OR (95% CI) for offspring’s ALT elevation					
	No. (%) of offspring with ALT elevation	Unadjusted	*p*	Adjusted^a^	*p*	Adjusted^b^	*p*
Parents without MetS	234/2939 (8.33)	1.00		1.00		1.00	
Father with MetS	107/920 (12.72)	1.60 (1.21–2.12)	<0.001	1.59 (1.20–2.11)	0.001	1.21 (0.83–1.74)	0.320
Mother with MetS	50/431 (12.39)	1.56 (1.08–2.24)	0.018	1.57 (1.08–2.27)	0.017	0.84 (0.52–1.36)	0.483
Both parents with MetS	18/139 (13.99)	1.79 (0.98–3.25)	0.056	1.88 (1.03–3.44)	0.040	1.12 (0.52–2.38)	0.777
Parents without ALT elevation	214/2821 (8.22)	1.00		1.00		1.00	
Father with ALT elevation	98/790 (12.53)	1.60 (1.21–2.11)	<0.001	1.56 (1.17–2.08)	0.002	1.54 (1.12–2.12)	0.007
Mother with ALT elevation	100/724 (14.63)	1.91 (1.41–2.60)	<0.001	1.95 (1.43–2.67)	<0.001	1.90 (1.35–2.67)	<0.001
Both parents with ALT elevation	35/168 (23.15)	3.36 (2.16–5.23)	<0.001	2.93 (1.76–4.88)	<0.001	2.66 (1.60–4.42)	<0.001

^a^Adjusted for intake of carbohydrate, protein, and fat of the offspring and the parents.

^b^Adjusted for offspring’s age, sex, BMI SDS, and intake of carbohydrate, protein, and fat and the parents’ BMI and intake of carbohydrate, protein, and fat.

OR, odds ratio; CI, confidence interval; ALT, alanine aminotransferase; MetS, metabolic syndrome; BMI, body mass index; SDS, standard deviation score.

## 4. Discussion

Our results demonstrate an association between parental MetS and elevated liver transaminases and offspring’s MetS and elevated liver transaminases. Moreover, parental MetS was associated with ALT elevation as well as MetS in their children. The relationship between mothers and descendant was stronger than that between fathers and their children. In addition, nutritional factors were closely related between parents and descendant. Among the nutritional factors, higher protein intake was related to ALT elevation. Additionally, older age, male sex, and higher BMI were associated with MetS and ALT elevation. The association of parental MetS and ALT elevation with descendant was significant, even after adjusting for nutritional factors. Moreover, parental ALT elevation was positively associated with descendant, even after adjusting for age and BMI. In addition, the relationship between parental MetS and ALT elevation with their children was more prominent in youths with normal BMI or overweight compared than in those with obesity. These findings suggest a genetic link between parents and descendant. Beyond genetics, epigenetic modifications can play a pivotal role by altering gene expression without changing the DNA sequence itself, potentially passing on metabolic risks to the offspring (26). Additionally, the transmission or shared characteristics of the gut microbiome between parents and their children might also influence metabolic health, considering its profound role in nutrient processing, inflammation, and other metabolic processes (27).

Elevated liver transaminases was closely related to MetS as well as each metabolic component among the youth in our study. MetS and NAFLD share insulin resistance as an important factor in pathogenesis (18). Insulin resistance promotes hepatic *de novo* lipogenesis by activating sterol regulatory element–binding protein 1 and lipid accumulation by inactivating Forkhead box protein A2, a transcription factor that promotes fatty acid oxidation in the liver (28, 29). Meanwhile, the secretion of hepatokine and metabolites related to lipid metabolism and insulin sensitivity is altered in NAFLD, which induces insulin resistance and MetS (30). A cross–sectional study reported that WC, blood pressure, and triglycerides increased, and HDL–C decreased with an increase in NAFLD grade among youth (31). A retrospective study reported that the odds ratio of NAFLD for MetS was 2.65 among children with obesity (32).

In our study, older age, male sex, BMI SDS, and obesity were found to be positively associated with MetS and elevated liver transaminases. Moreover, the association of elevated liver transaminases with age, male sex, and BMI SDS and that of MetS with BMI SDS were significant even after adjusting for nutritional factors. In a population–based study, the prevalence of NAFLD was 1.5–fold higher in participants aged 16–18 years than in those aged 10–12 years, while obesity was associated with a five–fold risk of NAFLD (7). In addition, the prevalence of NAFLD was two–fold higher in boys than in girls. In a systematic review, the prevalence of MetS was 29.2% in children with obesity and 3.3% in the whole population, while the corresponding value was 5.6% among older age groups and 2.9% among younger age groups (33).

Among nutritional factors, protein intake was associated with elevated liver transaminases in our study. Diets rich in proteins can induce production of harmful intestinal metabolites by gut microbiome, which may exacerbate MetS and NAFLD (34). A population–based study reported that protein intake in men with NAFLD was higher than that in men without, and this was thought to be related to meat intake and intestinal metabolites that are harmful for metabolic health due to intestinal protein fermentation (35, 36). However, another study reported that NAFLD development was not significantly related to protein intake (37). Meanwhile, the association of protein intake, age, and BMI SDS with ALT elevation was demonstrated in the youth of parents without MetS or ALT elevation in our study. Therefore, attention to liver enzymes and dietary intake is required in older boys with obesity, even though their parents do not have NAFLD or MetS, although more studies are required to clarify the association between nutritional intake and pediatric NAFLD.

The association of nutritional intake between parents and descendant was strong in our study. Thus, sharing meals and nutrition habits might affect the relationship between MetS and NAFLD of children and their parents. A meta–analysis reported that parental food preferences were significantly correlated with children’s preferences (38). In another meta–analysis, family based interventions improved the BMI of obese children (39). A population–based study reported that parents’ restriction on unhealthy food was negatively associated with the intake of unhealthy food in children (40). Parental nutrition can affect the descendant even during the prenatal period. Brion et al. (41) reported that the association between the descendant’s dietary habits and maternal dietary habits was stronger prenatally than postnatally. Moreover, a systematic review reported that the periconceptional nutritional status of parents could increase the risk of descendant MetS through epigenetic imprinting (42). Therefore, the assessment of familial nutritional status is required for the risk management of MetS and NAFLD in children, especially those with parents with MetS and NAFLD.

In our study, the association between parental MetS and elevated liver transaminases and those of their children was significant even after adjusting for nutritional intake. Moreover, parental elevated liver transaminases was associated with those of their even after adjusting for age, sex, BMI, and nutritional factors. These findings support the role of genetic links between MetS and elevated liver transaminases in parents and descendant. Several genes, including adiponutrin/patatin–like phospholipase domain–containing 3 and glucokinase regulatory protein, are known to be associated with both MetS and NAFLD (43). Adiponutrin/patatin–like phospholipase domain–containing 3 encodes adiponectin, which is expressed in both the liver and adipose tissue and has lipogenesis and lipolytic activity. Thus, limited triglyceride hydrolysis by the adiponutrin/patatin–like phospholipase domain–containing 3 rs738409 variant increases lipogenic activity. In a cohort study, glucokinase regulatory protein, a gene related to the regulation of glucokinase, was associated with triglyceride elevation and NAFLD among youths with obesity in a cohort study (44).

In our study, the association of children with mothers was stronger than that of fathers, and the ORs of children with both parents with MetS or elevated liver transaminases for children with MetS and elevated liver transaminases were higher than those with one parent with MetS and elevated liver transaminases. In a cohort study, the odds ratio of hepatic steatosis in at least one parent with hepatic steatosis was 2.0, whereas that of both parents with hepatic steatosis was 6.4 (8). In a meta–analysis study, the association of pediatric MetS with maternal MetS was stronger than that of paternal MetS, and that with both parents was strongest (45). The stronger lifestyle associations of children with mothers than those with fathers might be related to this difference. A cohort study reported that the association of children’s dietary intake with maternal dietary intake was stronger than that of paternal dietary intake (41). A Korean population–based study reported that adolescent physical activity is significantly associated with maternal physical activity (46).

The stronger association of offspring’s MetS and elevated liver transaminases with their parent in participants with normal BMI compared to those with obesity in our study is thought to be due to the strong association between obesity and MetS and NAFLD (7, 12, 33). In other words, the proportion of genetic effects on MetS and NAFLD may be higher in children without obesity. In an adult cohort study, parental hepatic steatosis is associated with hepatic steatosis in the descendant, even after adjusting for BMI, age, sex, alcohol, insulin resistance, and lipid–lowering agents (8). Therefore, attention to MetS and NAFLD is required, even in normal–weight children, if their parents have MetS and/or NAFLD.

This study has some limitations. First, this was a cross–sectional study limited to Koreans. Second, confounding factors such as physical activity, puberty, gut microbiome, and epigenetic modification were not considered. Third, liver biopsy and imaging studies were not performed for diagnosis of NAFLD or MAFLD because information on liver biopsy or imaging studies is not provided in the KNHANES. Fourth, genetic studies were not performed because the KNHANES does not include information on genetic evaluation. Fifth, KNHANES is not a complete enumeration, thus we analyzed the data with complex sampling design considering sampling weight to overcome selection bias.

## 5. Conclusion

This study showed a significant correlation between the MetS and elevated liver transaminase statuses of parents and their children, even after adjusting for nutritional factors, and a correlation between MetS and elevated liver transaminases in youths. In children, protein intake was associated with elevated liver transaminases, whereas the parental nutritional intake was associated with the child’s nutritional intake. The correlation between parental MetS and elevated liver transaminase status with that of children was more apparent in youths with a normal BMI or overweight than in those with obesity. These findings emphasize the role of genetic links and lifestyle in pediatric MetS, MAFLD, and NAFLD. Therefore, close monitoring of metabolic risk factors, including liver enzymes and dietary assessment, is required among the children of parents with MetS and/or NAFLD, even among those without obesity. Further studies to investigate the risk of pediatric MetS, NAFLD, and MAFLD by integrating genetic background and lifestyle factors are required to guide the management of future risks of cardiovascular disease and liver disease.

## Data availability statement

The raw data supporting the conclusions of this article will be made available by the authors, without undue reservation.

## Ethics statement

The studies involving humans were approved by the Institutional Review Board of Yonsei University Gangnam Severance Hospital (3-2022-0242). The studies were conducted in accordance with the local legislation and institutional requirements. Written informed consent for participation in this study was provided by the participants’ legal guardians/next of kin.

## Author contributions

KS and HWC had the idea and designed the study, had full access to all the data in the study, take responsibility for the integrity of the data and the accuracy of the data analysis, critically revised the manuscript for important intellectual content, and gave final approval for the version to be published. KS, JY, and HSL drafted the manuscript and did the analysis. JO, SK, ML, JS, AK, and H-SK take the responsibility for double check of the data analysis. All authors agree to be accountable for all aspects of the work in ensuring that questions related to the accuracy or integrity of any part of the work are appropriately resolved.
