# Supplementary material for: Parental metabolic syndrome and elevated liver transaminases are risk factors for offspring, even in children and adolescents with a normal body mass index
Source: Front Nutr. 2023 Oct 24;10:1166244. doi: 10.3389/fnut.2023.1166244 (PMC10627857; doi:10.3389/fnut.2023.1166244)
Supplement: Supplementary file 1 [file Data_Sheet_1.docx]

Supplementary Material

Parental Metabolic Syndrome and Elevated Liver Transaminases are Risk Factors for Offspring, Even in Children and Adolescents with a Normal Body Mass Index

**Kyungchul Song^1^, Juyeon Yang^2^, Hye Sun Lee^2^, Jun Suk Oh^3^, Sujin Kim^1^, Myeongseob Lee^1^, Junghwan Suh^1^, Ahreum Kwon^1^, Ho-Seong Kim^1^, Hyun Wook Chae^1*^**

^1^Department of Pediatrics, Yonsei University College of Medicine, Seoul, South Korea

^2^Biostatistics Collaboration Unit, Yonsei University College of Medicine, Seoul, South Korea

^3^Department of Pediatrics, Konyang University College of Medicine, Daejeon, South Korea

*** Correspondence:**Hyun Wook Chae

E-mail address: [hopechae@yuhs.ac](mailto:hopechae@yuhs.ac)

**Supplementary Table 1** ORs and 95% CIs for offspring’s MetS and ALT elevation

|  | Univariable | | | | Multivariable^a^ | | | | Multivariable^b^ | | | | Multivariable^c^ | | | |
| --- | --- | --- | --- | --- | --- | --- | --- | --- | --- | --- | --- | --- | --- | --- | --- | --- |
|  | MetS | | ALT elevation | | MetS | | ALT elevation | | MetS | | ALT elevation | | MetS | | ALT elevation | |
|  | OR (95% CI) | p | OR (95% CI) | p | OR (95% CI) | p | OR (95% CI) | p | OR (95% CI) | p | OR (95% CI) | p | OR (95% CI) | p | OR (95% CI) | p |
| Age | 1.13 (1.02‒1.25) | 0.022 | 1.08 (1.03‒1.14) | 0.003 | 1.06 (1.00‒1.13) | 0.040 | 1.03 (0.91‒1.16) | 0.666 |  |  |  |  | 1.04(0.92‒1.17) | 0.535 | 1.06(1.01‒1.13) | 0.028 |
| Sex, male | 1.52 (0.90‒2.55) | 0.115 | 2.73 (2.10‒3.54) | <0.001 | 2.99 (2.24‒4.00) | <0.001 | 1.22 (0.63‒2.33) | 0.558 |  |  |  |  | 1.38(0.66‒2.89) | 0.399 | 2.98(2.19‒3.07) | <0.001 |
| BMI SDS | 4.01 (3.31‒4.85) | <0.001 | 2.35 (2.11‒2.61) | <0.001 | 2.29 (2.05‒2.56) | <0.001 | 3.69 (3.00‒4.54) | <0.001 |  |  |  |  | 3.73(3.00‒4.64) | <0.001 | 2.31(2.07‒2.59) | <0.001 |
| Carbohydrate, g | 1.00 (0.98‒1.02) | 0.801 | 1.01 (1.00‒1.02) | 0.116 |  |  |  |  | 1.00 (0.98‒1.03) | 0.846 | 1.00 (0.99‒1.01) | 0.972 | 1.00(0.98‒1.03) | 0.883 | 0.99(0.98‒1.01) | 0.373 |
| Protein, g | 0.99 (0.93‒1.06) | 0.767 | 1.05 (1.02‒1.08) | <0.001 |  |  |  |  | 1.02 (0.94‒1.10) | 0.724 | 1.08 (0.04‒1.13) | <0.001 | 0.99(0.89‒1.11) | 0.848 | 1.05(1.00‒1.10) | 0.041 |
| Fat, g | 0.95 (0.86‒1.04) | 0.244 | 1.02 (0.99‒1.06) | 0.236 |  |  |  |  | 0.92 (0.81‒1.05) | 0.223 | 0.96 (0.91‒1.01) | 0.090 | 0.96(0.84‒1.10) | 0.556 | 0.97(0.92‒1.02) | 0.223 |
| MetS |  |  | 8.91 (5.16‒15.38) | <0.001 |  |  | 1.56 (0.80‒3.02) | 0.190 |  |  | 8.98 (5.22‒15.46) | <0.001 |  |  | 1.52(0.79‒2.91) | 0.212 |
| ALT elevation | 8.91 (5.16‒15.38) | <0.001 |  |  | 1.61 (0.75‒3.47) | 0.220 |  |  | 8.96 (5.20‒15.44) | <0.001 |  |  | 1.51(0.71‒3.22) | 0.287 |  |  |

P value determines using logistic regression.

^a^Multivariable logistic regression analyses including the dependent variable as MetS or ALT elevation and the independent variables including age, sex, BMI SDS, MetS, and ALT elevation.

^b^Multivariable logistic regression analyses including the dependent variable as MetS or ALT elevation and the independent variables including carbohydrate, protein, fat, MetS, and ALT elevation.

^c^Multivariable logistic regression analyses including the dependent variable as MetS or ALT elevation and the independent variables including age, sex, BMI SDS, carbohydrate, protein, fat, MetS, and ALT elevation.

OR, odds ratio; CI, confidence interval; MetS, metabolic syndrome; ALT, alanine aminotransferase; BMI, body mass index; SDS, standard deviation score.

**Supplementary Table 2** Characteristics of the offspring of parents without MetS or ALT elevation

|  | Offspring without Mets  (n = 2,130) | Offspring with Mets  (n = 28) | *p* | Offspring without ALT elevation  (n = 2,007) | Offspring with ALT elevation  (n = 151) | *p* |
| --- | --- | --- | --- | --- | --- | --- |
| BMI SDS | -0.21 (0.03) | 2.52 (0.16) | <0.001 | -0.27 (0.03) | 1.10 (0.13) | <0.001 |
| BMI percentile, % |  |  | <0.001 |  |  | <0.001 |
| Normal | 84.81% (0.94) | 0.00% (0.00) |  | 86.24% (0.91) | 50.22% (4.59) |  |
| Overweight | 8.55% (0.69) | 2.98% (2.39) |  | 8.04% (0.69) | 13.61% (3.36) |  |
| Obesity | 6.64% (0.69) | 97.02% (2.39) |  | 5.72% (0.63) | 36.17% (4.39) |  |
| WC | 68.43 (0.24) | 91.68 (1.33) | <0.001 | 67.88 (0.24) | 79.87 (1.03) | <0.001 |
| Central obesity, % | 6.58% (0.69) | 100.00% (0.00) | <0.001 | 5.97% (0.68) | 32.94% (4.26) | <0.001 |
| Systolic BP | 106.58 (0.28) | 117.77 (2.42) | <0.001 | 106.32 (0.29) | 112.04 (1.09) | <0.001 |
| Diastolic BP | 65.97 (0.23) | 71.48 (2.72) | 0.042 | 65.84 (0.24) | 68.67 (0.82) | <0.001 |
| Glucose, mg/dL | 89.68 (0.22) | 93.94 (2.14) | 0.047 | 89.61 (0.21) | 91.42 (1.36) | 0.184 |
| HDL-C, mg/dL | 51.94 (0.28) | 37.97 (0.95) | <0.001 | 51.94 (0.29) | 49.08 (0.95) | 0.003 |
| Triglycerides, mg/dL | 82.74 (1.38) | 187.42 (17.32) | <0.001 | 81.11 (1.25) | 123.82 (9.40) | <0.001 |
| AST, IU/L | 19.12 (0.16) | 25.36 (3.39) | 0.066 | 18.29 (0.12) | 30.41 (1.26) | <0.001 |
| ALT, IU/L | 14.13 (0.30) | 35.23 (7.43) | 0.005 | 12.01 (0.11) | 44.08 (2.55) | <0.001 |
| MetS, % |  |  |  | 0.81% (0.24) | 10.47% (3.07) | <0.001 |
| ALT elevation, % | 6.93% (0.68) | 51.56% (10.85) | <0.001 |  |  |  |
| Energy intake, kcal | 2135.83 (22.27) | 2134.58 (183.31) | 0.995 | 2123.56 (22.53) | 2284.10 (97.46) | 0.108 |
| Carbohydrate intake, g | 324.00 (3.42) | 325.63 (28.52) | 0.955 | 323.11 (3.50) | 335.20 (13.77) | 0.397 |
| Protein intake, g | 77.43 (1.02) | 83.71 (8.54) | 0.462 | 76.38 (0.97) | 91.42 (5.85) | 0.011 |
| Fat intake, g | 56.35 (1.23) | 52.35 (11.41) | 0.724 | 56.08 (1.25) | 58.88 (4.20) | 0.501 |

Continuous variables are presented as the mean (standard error) and categorical data as the percentage (standard error).

MetS, metabolic syndrome; ALT, alanine aminotransferase; BMI, body mass index; SDS, standard deviation score; WC, waist circumference; BP, Blood pressure; HDL-C, high-density lipoprotein cholesterol; AST, aspartate aminotransferase; ALT, alanine aminotransferase.

**Supplementary Table 3** Linear regression of offspring's metabolic components and nutrition with parental metabolic components and nutrition

|  | Beta (standard error) | *p* | r^2^ |
| --- | --- | --- | --- |
| Father |  |  |  |
| BMI for offspring's BMI SDS | 0.10 (0.01) | <0.001 | 0.061 |
| WC for offspring's WC | 0.06 (0.01) | <0.001 | 0.839 |
| Systolic BP for offspring's systolic BP | 0.11 (0.01) | <0.001 | 0.226 |
| Diastolic BP for offspring's diastolic BP | 0.11 (0.02) | <0.001 | 0.137 |
| Glucose for offspring's glucose | 0.04 (0.01) | <0.001 | 0.066 |
| HDL-C for offspring's HDL-C | 0.24 (0.02) | <0.001 | 0.162 |
| Triglycerides for offspring's triglycerides | 0.04 (0.01) | <0.001 | 0.089 |
| AST for offspring's AST | 0.02 (0.01) | 0.013 | 0.091 |
| ALT for offspring's ALT | 0.03 (0.01) | 0.029 | 0.137 |
| Energy for offspring's energy | 0.12 (0.02) | <0.001 | 0.110 |
| Carbohydrate for offspring's carbohydrate | 0.15 (0.02) | <0.001 | 0.096 |
| Protein for offspring's protein | 0.17 (0.03) | <0.001 | 0.111 |
| Fat for offspring's fat | 0.20 (0.03) | <0.001 | 0.088 |
| Mother |  |  |  |
| BMI for offspring's BMI SDS | 0.11 (0.01) | <0.001 | 0.084 |
| WC for offspring's WC | 0.08 (0.01) | <0.001 | 0.841 |
| Systolic BP for offspring's systolic BP | 0.11 (0.01) | <0.001 | 0.225 |
| Diastolic BP for offspring's diastolic BP | 0.13 (0.02) | <0.001 | 0.142 |
| Glucose for offspring's glucose | 0.11 (0.06) | 0.063 | 0.113 |
| HDL-C for offspring's HDL-C | 0.26 (0.02) | <0.001 | 0.193 |
| Triglycerides for offspring's triglycerides | 0.13 (0.02) | <0.001 | 0.098 |
| AST for offspring's AST | 0.07 (0.02) | <0.001 | 0.094 |
| ALT for offspring's ALT | 0.09 (0.04) | 0.036 | 0.139 |
| Energy for offspring's energy | 0.25 (0.03) | <0.001 | 0.127 |
| Carbohydrate for offspring's carbohydrate | 0.23 (0.03) | <0.001 | 0.113 |
| Protein for offspring's protein | 0.27 (0.03) | <0.001 | 0.115 |
| Fat for offspring's fat | 0.35 (0.03) | <0.001 | 0.110 |

Values are presented as regression coefficient (standard errors). Beta means regression coefficient. r^2^ means coefficient of determination.

BMI, body mass index; SDS, standard deviation score; WC, waist circumference; BP, Blood pressure; HDL-C, high-density lipoprotein cholesterol; ALT, alanine aminotransferase.
